# An App-Based Parenting Program to Promote Healthy Energy Balance–Related Parenting Practices to Prevent Childhood Obesity: Protocol Using the Intervention Mapping Framework

**DOI:** 10.2196/24802

**Published:** 2021-05-14

**Authors:** Levie T Karssen, Jacqueline M Vink, Carolina de Weerth, Roel C J Hermans, Carina P M de Kort, Stef PJ Kremers, Emilie L M Ruiter, Junilla K Larsen

**Affiliations:** 1 Behavioural Science Institute Radboud University Nijmegen Netherlands; 2 Donders Institute for Brain, Cognition and Behaviour Radboud University Medical Center Nijmegen Netherlands; 3 NUTRIM School of Nutrition and Translational Research in Metabolism Maastricht University Medical Center Maastricht Netherlands; 4 Netherlands Nutrition Centre The Hague Netherlands; 5 Institute of Health Studies HAN University of Applied Sciences Nijmegen Netherlands; 6 Academic Collaborative Centre AMPHI Primary and Community Care Radboud University Medical Centre Nijmegen Netherlands

**Keywords:** childhood obesity, preventive intervention, parenting practices, energy-balance related behavior, socio-economic position, mHealth, behavior change, mobile phone

## Abstract

**Background:**

The family environment plays an important role in the development of children’s energy balance–related behaviors. As a result, parents’ energy balance–related parenting practices are important targets of preventive childhood obesity programs. Families with a lower socioeconomic position (SEP) may benefit from participating in such programs but are generally less well reached than families with a higher SEP.

**Objective:**

This paper describes the application of the Intervention Mapping Protocol (IMP) for the development of an app-based preventive intervention program to promote healthy energy balance–related parenting practices among parents of children (aged 0-4 years) with a lower SEP.

**Methods:**

The 6 steps of the IMP were used as a theory- and evidence-based framework to guide the development of an app-based preventive intervention program.

**Results:**

In step 1, behavioral outcomes for the app-based program (ie, children have a healthy dietary intake, sufficient sleep, and restricted screen time and sufficient physical activity) and sociocognitive (ie, knowledge, attitudes, and self-efficacy) and automatic (ie, habitual behaviors) determinants of energy balance–related parenting were identified through a needs assessment. In step 2, the behavioral outcomes were translated into performance objectives. To influence these objectives, in step 3, theory-based intervention methods were selected for each of the determinants. In step 4, the knowledge derived from the previous steps allowed for the development of the app-based program *Samen Happie!* through a process of continuous cocreation with parents and health professionals. In step 5, community health services were identified as potential adopters for the app. Finally, in step 6, 2 randomized controlled trials were designed to evaluate the process and effects of the app among Dutch parents of infants (trial 1) and preschoolers (trial 2). These trials were completed in November 2019 (trial 1) and February 2020 (trial 2).

**Conclusions:**

The IMP allowed for the effective development of the app-based parenting program *Samen Happie!* to promote healthy energy balance–related parenting practices among parents of infants and preschoolers. Through the integration of theory, empirical evidence, and data from the target population, as well as the process of continued cocreation, the program specifically addresses parents with a lower SEP. This increases the potential of the program to prevent the development of obesity in early childhood among families with a lower SEP.

**Trial Registration:**

Netherlands Trial Register NL6727, https://www.trialregister.nl/trial/6727; Netherlands Trial Register NL7371, https://www.trialregister.nl/trial/7371.

## Introduction

### Background

Although childhood obesity rates have been reported to stabilize in many developed countries [1], the prevalence of overweight and obesity in young children is still high. In general, 8% of Dutch children around the age of 2 years were overweight or obese in 2018 [2]. However, these rates were considerably higher among children from families with a lower socioeconomic position (SEP) [2]. In addition, a growing body of research indicates that plateauing prevalence rates are predominantly evident among groups with a higher SEP [3]. In groups with a lower SEP, the rates are still rising [4], implying an increase in socioeconomic health disparities. To reduce SEP disparities in childhood obesity, preventive intervention programs should promote healthy energy balance–related behaviors (EBRBs; ie, dietary intake, sleep, physical activity, and screen time) before children have developed fixed patterns of EBRBs [5]. As patterns of EBRBs, such as dietary intake and screen time, develop in the first years of life [6], infancy and preschool (0-4) years present a critical window for childhood obesity prevention.

Parents are considered the main agents of change in effective preventive intervention programs for childhood obesity, especially in the first years of life [7]. Parents’ behaviors, particularly their energy balance–related parenting practices (ie, energy balance–related, specific, discrete, and observable acts of parenting [8]), may significantly influence their children’s EBRBs and body weight [9,10]. Recent reviews have shown that promoting responsive feeding guidance to teach maternal awareness and attention to children’s hunger and satiety cues can support *normal* child body weight development [11]. Moreover, universal preventive intervention programs targeting early feeding and positive parenting skills, including programs that target sleep, show similar effects on the child’s body weight [12]. In general, current evidence supports starting with promoting responsive feeding and parenting during infancy and incorporating the promotion of structure and rule setting in early childhood [13]. For physical activity parenting, support has been found for the potential importance of parental support and parents’ own behaviors and role modeling [14,15]. However, it should be noted that only a few preventive intervention programs have targeted physical activity parenting [14]. More generally, preventive intervention programs for early childhood obesity that target parenting practices with respect to all relevant child EBRBs are scarce [12]. Moreover, parent-focused prevention programs have been mostly universal (ie, population based) in nature [12,16]. For instance, 3 of 4 parent-focused EPOCH (Early Prevention of Obesity in Children) trials that commenced by early infancy were universal programs [17]. Our parent-focused program to prevent early childhood obesity adds to the literature by addressing parenting practices with respect to all the important child EBRBs [12,18] while simultaneously applying selective prevention to the subgroup of parents with a lower SEP, who generally display more problematic energy balance–related parenting compared with other SEP groups [19].

In addition, our program uses an innovative approach to address 2 frequently reported limitations of traditional (parent focused) obesity prevention programs: the costly, time-intensive face-to-face setting [16,20] and difficulties in reaching people with a lower SEP [21]. To successfully target and reach people with a lower SEP, intervention programs should address both practical (eg, time constraints, lack of transportation, and inflexible working hours [22-24]) and attitudinal (eg, irrelevant and not engaging program elements [25]) barriers for participation [26]. Practical barriers for people with a lower SEP could (at least partly) be overcome by delivering interventions through smartphones, including mobile apps [21,27]. Furthermore, attitudinal barriers can also be overcome because app-based interventions allow for options to increase program engagement through the presentation of bite-sized information in plain language that is accompanied by appealing visuals and through the possibility of personalizing the intervention and the ability for users to monitor their behavior [28,29]. For these reasons, app-based intervention programs have the potential to be cost-effective and reduce the gap in socioeconomic health disparities [29,30]. The apps used in these programs need to be high quality (eg, in terms of engagement, esthetics, and information quality [31]) and encourage behavior change (eg, providing knowledge and information as well as prompting goal setting and planning [32]). To achieve this, app-based interventions should be based on evidence, grounded in behavior change theory, and incorporate the needs and wishes of the target audience through formative research [33].

### Objectives

Therefore, we developed the app-based prevention program *Samen Happie!* using the Intervention Mapping Protocol (IMP). The IMP is a widely used, standardized intervention planning format that helps intervention developers to incorporate empirical findings from the literature, effective behavioral change methods and their practical applications, and data collected in a representative population [34]. Although it is not specific to app-based program development, the IMP has been successfully applied to the development of digital (including mobile) interventions for youth health promotion [35-41]. The central goal of the app-based preventive parenting program *Samen Happie!* is to stimulate healthy energy balance–related parenting practices to prevent early childhood obesity among children of families with a lower SEP. In this paper, we inform readers about the development of the *Samen Happie!* program based on the 6 steps of intervention mapping.

The Dutch title of the app-based parenting program (*Samen Happie!*) will be used throughout this paper. A possible translation is “Happy Together,” but this does not reflect the play on words the title indicates in Dutch.

## Methods

### Overview

The *Samen Happie!* program was developed through the 6 iterative and nonlinear steps of the IMP: (1) conducting a needs assessment; (2) preparing matrices of change objectives; (3) selecting theoretical methods and practical strategies; (4) developing the intervention program; (5) planning for adoption, implementation, and sustainability; and (6) planning the program evaluation [34]. We collaborated with a workgroup consisting of potential program implementers (ie, youth health care professionals of community health services) and users (ie, parents of young children with a lower SEP). Importantly, for the development and evaluation of the program, we asked only one parent-child dyad per family to participate, thereby focusing on the primary caregiver. In this section, the main tasks of each step of the IMP are explained and, when relevant, the role of the workgroup is exemplified. The outcomes of the IMP, including the choices and actions during each step, are described in the Results section.

### Step 1: Conducting a Needs Assessment

The first step of the IMP was to conduct a needs assessment of our target group (ie, parents of children aged 0 to 4 years with a lower SEP) to build a logic model of the health problem [34]. Our needs assessment included a literature search, focus groups with parents with a lower SEP (N=16 mothers in total), and discussions with youth health care professionals (N=6 professionals in total). In the focus groups, the hindering and facilitating factors for healthy parenting and parenting practices in difficult parenting situations were discussed, with a focus on parenting practices regarding food and dietary intake. The focus groups, which were conducted until saturation was reached, were audio recorded, transcribed, and coded for themes and concepts using ATLAS.ti (ATLAS.ti Scientific Software Development GmbH). The discussions with youth health care professionals served to explore key parenting-related themes and issues that existed among the target group and as a sounding board for concrete questions during program development. For example, frequently reported parenting problems, (parental adherence to) national guidelines regarding child EBRBs, and effective, practical strategies to increase healthy child EBRBs through energy balance–related parenting practices were discussed. To build the logic model (based on the PRECEDE [Predisposing, Reinforcing, and Enabling Constructs in Educational Diagnosis and Evaluation] framework [42]), we identified the quality-of-life indicators associated with the health problem, behavioral and environmental risk behaviors for the health problem, and determinants related to these behaviors. On the basis of the knowledge derived from this assessment, we selected behavioral outcomes for the program and formulated the program goal.

### Step 2: Preparing Matrices of Change Objectives

In the second step of the IMP, the performance objectives were defined for the behavioral outcomes specified in step 1. These performance objectives constituted behaviors that are expected to contribute to achieving the program goal when performed by the target group [34]. By crossing the performance objectives with the determinants selected in step 1, the change objectives were specified. These change objectives indicate which actions are required to modify the determinants of the behavioral outcomes and reach the performance objectives [34].

### Step 3: Selecting Theoretical Methods and Practical Strategies

The third step of the IMP evolved around the selection of theory- and evidence-based change methods to affect the determinants selected in step 1. We aimed to select a limited number of theoretical methods per determinant as interventions that use a small number of behavior change techniques are generally more effective for people with a lower SEP than interventions that use a larger number of techniques [43]. We then translated the selected methods into practical strategies through which they were delivered in the program [34].

### Step 4: Developing the Intervention Program

The fourth step of the IMP involved building the program themes and components and drafting, pretesting, and producing the program materials based on the information gathered in the previous steps [34]. The development of the app-based parenting program consisted of 3 phases. In phase 1, qualitative user research was conducted to assess parents’ (N=16 mothers) wishes regarding the content and functionality of the app. In phase 2, a prototype of the app structure, functionalities, content, and visual design was constructed in continuous cocreation with parents (N=4 mothers) and youth health care professionals (N=3) and pretested by parents (N=16 mothers). Finally, phase 3 involved building the final version of the app. For the development of the app, we collaborated with Dio Agency, an agency specialized in deploying software design to facilitate behavioral change.

### Step 5: Planning for Adoption, Implementation, and Sustainability

The fifth step of the IMP involved the identification of potential program users (eg, implementers or adopters) and the design of a program implementation plan [34]. We planned to make the app-based intervention available free of charge after program evaluation; nevertheless, the excess supply of health-related apps minimizes the chances that parents will spontaneously find and download the *Samen Happie!* app. This is one of the reasons that digital health programs in particular need a delivery system (ie, a program adopter) to get the program to its intended participants [34].

### Step 6: Planning the Program Evaluation

The sixth and last step of the IMP involved the development of a program evaluation plan [34]. We developed both process and effect evaluation plans to evaluate the quality of the implementation and the effectiveness of the preventive intervention program.

## Results

### Step 1: Conducting a Needs Assessment

Our logic model of the health problem is presented in Multimedia Appendix 1. The model displays the quality-of-life indicators (eg, cardiovascular diseases, depression, and risk of obesity in adulthood [44-46]) as correlates of health problems (ie, early childhood obesity). Moreover, it shows behavioral (eg, child dietary intake) and environmental (eg, unhealthy home environment) risk factors for health problems and determinants related to these factors (ie, parents’ knowledge about healthy dietary intake). The parental determinants impact child risk behaviors through energy balance–related parenting practices. The child energy balance–related risk behaviors, role of parents, and determinants of healthy parenting are described hereafter.

#### Child Risk Behaviors and the Role of Parents

Ample research has established the intake of energy-rich foods and sugar-sweetened drinks [47-49] and longer screen time and shorter sleep duration [50-53] as key modifiable risk behaviors of childhood obesity. Notably, these unhealthy EBRBs are more common among children from lower SEPs than among those from families with a higher SEP [54-56]. This led us to select the following behavioral outcomes of our preventive intervention program: (1) children have a healthy dietary intake (ie, food and drinks), (2) children get sufficient sleep, and (3) children have a healthy balance between screen time and physical activity. Especially early in life, child EBRBs are largely shaped by parents and their parenting practices [57]. Hence, the overall program goal was to improve child EBRBs (ie, dietary intake, sleep, physical activity, and screen time) and subsequent body weight through the stimulation of healthy energy balance–related parenting practices.

#### Energy Balance–Related Parenting Practices

Parenting practices are broadly divided into 3 overarching dimensions of food parenting [10] that can also be observed in a wider range of energy balance–related parenting behaviors [58-62], namely, coercive control (ie, the use of pressure and dominance to control child behavior, such as restriction and threats [63]), structure (ie, the use of noncoercive forms of control, such as rules and routines [63]), and autonomy support (ie, the facilitation of children’s independence, for instance through responsive feeding and praise [64]). Studies have shown that structured and autonomy-supportive parenting practices are mostly related to favorable child energy balance–related outcomes, whereas coercive controlling practices show unfavorable associations with children’s EBRBs and body weight [61,65,66]. However, notably, as compared with parents with a higher SEP, parents with a lower SEP are more likely to use coercive control [56,67-72] and less likely to use structure-related [59,73-76] and autonomy-supportive parenting practices [70,77]. Hence, it is pivotal that preventive intervention programs for childhood obesity, particularly those targeting parents with a lower SEP, promote structure-related and autonomy-supportive practices and discourage coercive controlling parenting practices regarding children’s EBRBs.

#### General Parenting and Parental Well-being

Both general parenting and parental well-being may moderate the associations between parental energy balance–related parenting practices and child EBRBs. With respect to general parenting (ie, the broader emotional climate in which specific parenting practices are performed [78]), research has, for instance, demonstrated that the prospective associations between parental encouragement and covert control (eg, food availability) and dietary intake were stronger among children who were exposed to a positive parenting style [79]. Hence, desirable (ie, structured and autonomy-supportive) parenting practices performed in an authoritative parenting climate (ie, characterized by both demandingness and responsiveness [80]) might produce the largest intervention effects [9,59,81]. Moreover, research found reciprocal relationships between parental mental well-being and parenting, indicating that positive well-being among parents relates to higher parenting self-efficacy and more beneficial parenting practices [82]. Thus, improving parental well-being and stimulating authoritative parenting appear to be promising conditions for improving child EBRBs. Examining these moderators may not only provide more insight into potential differential intervention effects for subgroups of parents but it could also help identify eminent targets for future (personalized) obesity prevention programs.

#### Determinants of Healthy Parenting

For the selection of determinants, we were informed by the results of our focus groups and the empirical literature. Moreover, the I-Change model [83] was used as a basis to integrate influential theories on motivation and behavior change (ie, Theory of Planned Behavior [84], Social Cognitive Theory [85], the Transtheoretical Model [86], and the Health Belief Model [87]). The I-Change model explains how knowledge, attitudes, and self-efficacy play a role in a person’s motivation and intention to perform health behaviors. This model also considers the gap between the intention to perform a behavior and actually performing the behavior (ie, the intention-behavior gap [88]). In the *Samen Happie!* program, with respect to performing healthy parenting practices related to children’s EBRBs, the following determinants are targeted: parental knowledge, attitudes, self-efficacy, and habitual behavior (ie, habits).

#### Knowledge

Knowledge plays an important role in changing EBRBs and is a basic component of existing preventive intervention programs for childhood obesity [7]. It is particularly important to include remediation components in interventions for high-risk (eg, lower SEP) populations [89,90]. People with a lower SEP tend to have lower health literacy in general [91] and regarding healthy parenting in particular [92]. Illustratively, some parents in our focus groups held incorrect beliefs about the healthiness of drinks (eg, “Fruit juice is healthy because it contains vitamins”). Importantly, targeting knowledge may also indirectly change other sociocognitive determinants, including attitudes and self-efficacy [93].

#### Attitudes

Knowledge should be targeted by carefully considering the beliefs of the target group [94]. Some parents in our focus groups held negative attitudes toward specific energy balance–related parenting practices, such as providing water instead of sugar-sweetened drinks (eg, “Drinking water is for dogs”). These parental attitudes toward energy balance–related (parenting) behaviors can influence children’s behaviors, such as physical activity [95] and screen time [96,97]. When targeting attitudes, parents’ beliefs should be taken into consideration, as it has generally been acknowledged that it is difficult to change attitudes with a high affective component. Therefore, it is imperative to balance the stimulation of favorable attitudes about EBRBs with parents’ personal goals [98].

#### Self-efficacy

Self-efficacy refers to a parent’s beliefs in their capabilities to organize and execute a course of action (ie, performing energy balance–related parenting practices) in particular situations [99]. In general, children display more healthful behaviors if parents report higher self-efficacy [100], and improving parental self-efficacy also appears to be a promising approach to change young children’s EBRBs [101]. Enhancing self-efficacy might be especially important for parents with a lower SEP, as parents in our focus groups often felt insecure about their capabilities to employ healthy energy balance–related parenting practices (eg, sticking to a maximum amount of screen time).

#### Habits

Parents often report a discrepancy between what they intend to do in terms of energy balance–related parenting practices and what they actually do [88]. For instance, parents in our focus groups found it difficult to form healthy habits and routines, especially when unhealthy habits were already established (eg, eating in front of the television). Habits influence health behaviors [102], and parental energy balance–related habits may impact energy balance–related parenting practices [88]. One previous intervention program that trained parents to perform healthy parenting habits proved to be promising [103]. Thus, targeting the development of healthy habits may assist parents in the long-term adherence to newly developed energy balance–related parenting practices and may bridge the gap between parenting intentions and behaviors [88].

### Step 2: Preparing Matrices of Changes Objectives

Table 1 presents 3 examples of performance and change objectives for each behavioral outcome (ie, children have a healthy dietary intake, sufficient sleep, and restricted screen time and sufficient physical activity). Multimedia Appendix 2 [10] presents an overview of all performance objectives and change objectives specified for the app-based preventive intervention program.

**Table 1:** Change objectives for dietary intake, sleep, and restricted screen time and sufficient physical activity by crossing the determinants with the performance objectives.

Performance objectives			Determinants						
			Knowledge		Attitudes		Self-efficacy		Habits
**Dietary intake**									
	Parents apply clear rules about the consumption of healthy and unhealthy food products and drinks.	Parents explain how they can apply clear rules about the consumption of healthy and unhealthy food products or drinks.		Parents express positive feelings toward having clear rules for the consumption of healthy food products or drinks.		Parents express confidence in applying clear rules about the consumption of healthy and unhealthy food products or drinks.		Parents consistently apply clear rules about the consumption of healthy and unhealthy food products or drinks.	
	Parents act as a role model by eating or drinking healthy food or drinks themselves.	Parents explain how they can act as positive role models by eating or drinking healthy food or drinks themselves.		Parents express positive feelings toward acting as a role model by eating or drinking healthy food or drinks themselves.		Parents express confidence in acting as a role model by eating or drinking healthy food or drinks themselves.		Parents consistently act as a role model by eating or drinking healthy food or drinks themselves.	
	Parents praise their child when he or she eats healthy food products or drinks water.	Parents explain how they can praise their child when he or she eats healthy food products or drinks water.		Parents express positive feelings toward praising their child when he or she eats healthy food products or drinks water.		—^a^		—	
**Sleep**									
	Parents apply clear rules about bed times.	Parents explain how they can apply clear rules about bed times.		Parents express positive feelings toward applying clear rules about bed times.		—		—	
	Parents make use of bedtime routines.	Parents explain how they can make use of bedtime routines.		Parents express positive feelings toward making use of bedtime routines.		Parents express confidence in making use of bedtime routines.		Parents consistently make use of bedtime routines.	
	Parents ensure a safe and quiet sleep environment for their child.	Parents explain how they can ensure a safe and quiet sleep environment for their child.		Parents express positive feelings about ensuring a safe and quiet sleep environment for their child.		—		—	
**Restricted screen time and sufficient physical activity**									
	Parents apply clear rules about screen time.	Parents explain how they can apply clear rules about screen time.		Parents express positive feelings toward applying clear rules about screen time.		Parents express confidence in applying clear rules about screen time.		Parents consistently apply clear rules about screen time.	
	Parents facilitate activities without the use of screens.	Parents explain how they can facilitate activities without the use of screens.		Parents express positive feelings toward facilitating activities without the use of screens.		Parents express confidence in facilitating activities without the use of screens.		Parents consistently facilitate activities without the use of screens.	
	Parents encourage their child to be physically active (eg, playing outside).	Parents explain how they can encourage their child to be physically active.		Parents express positive feelings toward encouraging their child to be physically active.		—		—	

^a^Not all performance objectives were translated into change objectives.

### Step 3: Selecting Theoretical Methods and Practical Strategies

The theoretical methods we selected for each of the determinants (ie, knowledge, attitudes, self-efficacy, and habits) were derived from the taxonomies described by Kok et al [104] and Michie et al [105]. Table 2 presents an overview of the methods and examples of their practical application. This table shows that we included both methods involving the provision of information (eg, consciousness raising and persuasive communication) and those that target more automatic processes (eg, implementation intentions and self-nudging). Automatic, *nonconscious* methods might be particularly effective for people with a lower SEP as these methods are less dependent on literacy capabilities [106].

**Table 2:** Theoretical methods that were selected to address the determinants and examples of how these methods were applied in the app-based program.

Determinant and method			Definition		Example of practical application in the app
**Knowledge**					
	Consciousness raising	Giving information about the causes and consequences of a problem behavior and providing alternatives to substitute problem behaviors [104]		Parents are advised not to comfort or reward their children with food (ie, emotional and instrumental feeding). We explain that in doing so, children might learn to comfort themselves using food later in life or learn that they will be rewarded for demonstrating unwanted behavior. As alternatives for the problem behavior, we suggest to comfort or reward children with attention and affection (eg, giving a compliment, thumbs up, or hug)	
	Instruction on how to perform a behavior	Advise on how to perform the behavior [105]		We provide both simple and more elaborate advice on how to perform parenting behaviors. For instance, when parents want to encourage water consumption but their child is used to drinking fruit juice, we advise to gradually substitute parts of the fruit juice by water over the course of several weeks. A more elaborate advice is provided when parents want to decide which type of fruit juice is the healthier option. This advice involves 3 steps (ie, grab 2 drinks, turn them around and look at the food label, and pick the option with the least calories). For the second step (reading the food label), we advise parents to look at the amount of sugar and explain how they can calculate the amount of sugar per serving	
**Attitudes**					
	Persuasive communication	Guiding individuals toward the adoption of an idea, attitude, or action by using arguments or other means [104]		To encourage positive attitudes toward the consumption of water, we provide 3 benefits of drinking water (or tea): (1) it does not contain calories and contributes to a healthy body weight, (2) water and tea do not contain sugar, the teeth are not affected and cavities can be prevented, and (3) water supports the functioning of the body and can support learning and playing	
	Framing	Using gain-framed messages emphasizing the advantages of performing the healthy behavior [104]		We focused on providing gain-framed messages that emphasize the benefits for parents and/or children. Examples are “Did you know that you can save up to €150 euros per year if Maria drinks water instead of fruit juice?” and “Using a fixed bedtime routine can help Maria fall asleep faster”	
**Self-efficacy**					
	Verbal persuasion	Using messages that suggest that the participant possesses certain capabilities [104]		Before making an if-then plan (see *Implementation intentions*) to stimulate children’s water consumption, parents are asked whether they think drinking water is important and whether they feel confident in making their child drink (more) water. They answer on a scale from 0 (not important or confident at all) to 5 (very important or confident). On the basis of their answer, they receive an encouraging response (eg, “Many parents think they won’t be successful in getting their child to drink water. But you will be surprised to see that practice will eventually pay off. You can do it!”)	
	Action planning	Prompt detailed planning of performance of the behavior [105]		To regulate children’s screen time, parents are prompted to make a family screen time plan. For this plan, they first describe in which rooms screens can be used (eg, living room) and then at what times screens can be used (eg, before dinner). The plan should be focused on what *is* allowed, rather than what is *not* allowed (see also *Framing*). Parents are also prompted to write down this plan on paper and display it at a prominent place, and (if applicable) to discuss the plan with other caregivers	
**Habits**					
	Implementation intentions	Prompting making if-then plans that link situational cues with responses that are effective in attaining goals or desired outcomes [104]		Parents are prompted to make an if-then plan to stimulate their child’s physical activity. First, we explain what an if-then plan is and why it is important to make them. Next, 3 examples are presented (eg, “If we go outside together, then I will let Maria walk next to the stroller for a couple of minutes”). After that parents are asked to make their own plan by defining first *when* they can stimulate physical activity for their child (eg, if my child wakes up from her nap) and next *how* they will achieve this (eg, then I will play an active game with her)	
	Self-nudging	Making simple changes in the presentation of choice alternatives that make the desired choice the easy, automatic, or default choice [104]		We provide parents tips that can help make healthy eating at home the easy, automatic option: (1) buy mostly healthy food such as fruits and vegetables at the grocery store; (2) buy no or only a small amount of snacks, such as candy and chocolate; (3) display healthy foods in a way that they are easily noticed, eg, by presenting fruit in a bowl on the table; and (4) store unhealthy foods out of sight, for instance, keeping snacks at the back of the storage cabinet	

In addition to potentially important theoretical methods, the form in which an intervention is delivered is also a key ingredient in behavior change interventions [107] and might be even more crucial for groups with a lower SEP in terms of comprehensibility and engagement [108]. Considering the literacy capabilities of participants with a lower SEP, research has shown that people with literacy problems can remember written texts more easily when they are supported by (audio)visual aids [109,110]. Therefore, we included both written texts and supporting icons, images, videos, and voice-overs of important information in the app-based prevention program. Moreover, to ensure that the app was comprehensible for parents with varied literacy skills, all textual components were revised by a specialized organization to match language levels A2-B1 according to the Common European Framework of Reference [111]. In addition, the technological features of the app allowed us to tailor the program to individual participants, which might positively impact user engagement [112] and subsequent intervention retention. Illustratively, the app uses data about the name, sex, and birth date of the child (entered by the parent upon registration) to personalize texts with respect to names and pronouns (eg, “Set a good example for *Maria*. Try to eat healthy when *she* is around”). Finally, the data on children’s age were used to present parents with developmentally appropriate information via age-based modules (see *Step 4: Developing the Intervention Program*), which could enhance the perceived relevance of the information, thereby increasing engagement [113].

### Step 4: Developing the Intervention Program

In phase 1 of the app development process, the results from our focus groups and discussions with youth health care professionals informed the development of the prototype of the app and the way in which we tailored the materials to our target group. For instance, based on parents’ negative affective attitudes toward water consumption, we asked them whether they would provide their children tea (without sugar) as an alternative, and they affirmed that they would do so. Therefore, we drafted the app content with “water or tea,” instead of focusing entirely on water consumption. Other examples of content that we based on the suggestions of parents from our focus groups included providing information about saving money on groceries and tips to stimulate vegetable consumption.

In phase 2, the content of the preventive intervention program was drafted based on national health care guidelines, relevant literature on parenting practices in relation to child EBRBs, and previous intervention projects [103,114]. For example, youth health care professionals suggested using videoclips that were specially designed by the Dutch Child and Family Center (Centrum voor Jeugd en Gezin) to stimulate health literacy among young (low literate) parents. The pretest of the prototype of the app yielded, among others, the following suggestions: the option to enlarge images, the possibility of replaying the instruction clip, a summary of the most important information per level, and the option to reread a lesson. These suggestions were incorporated into the final version of the app.

**Figure 1:**
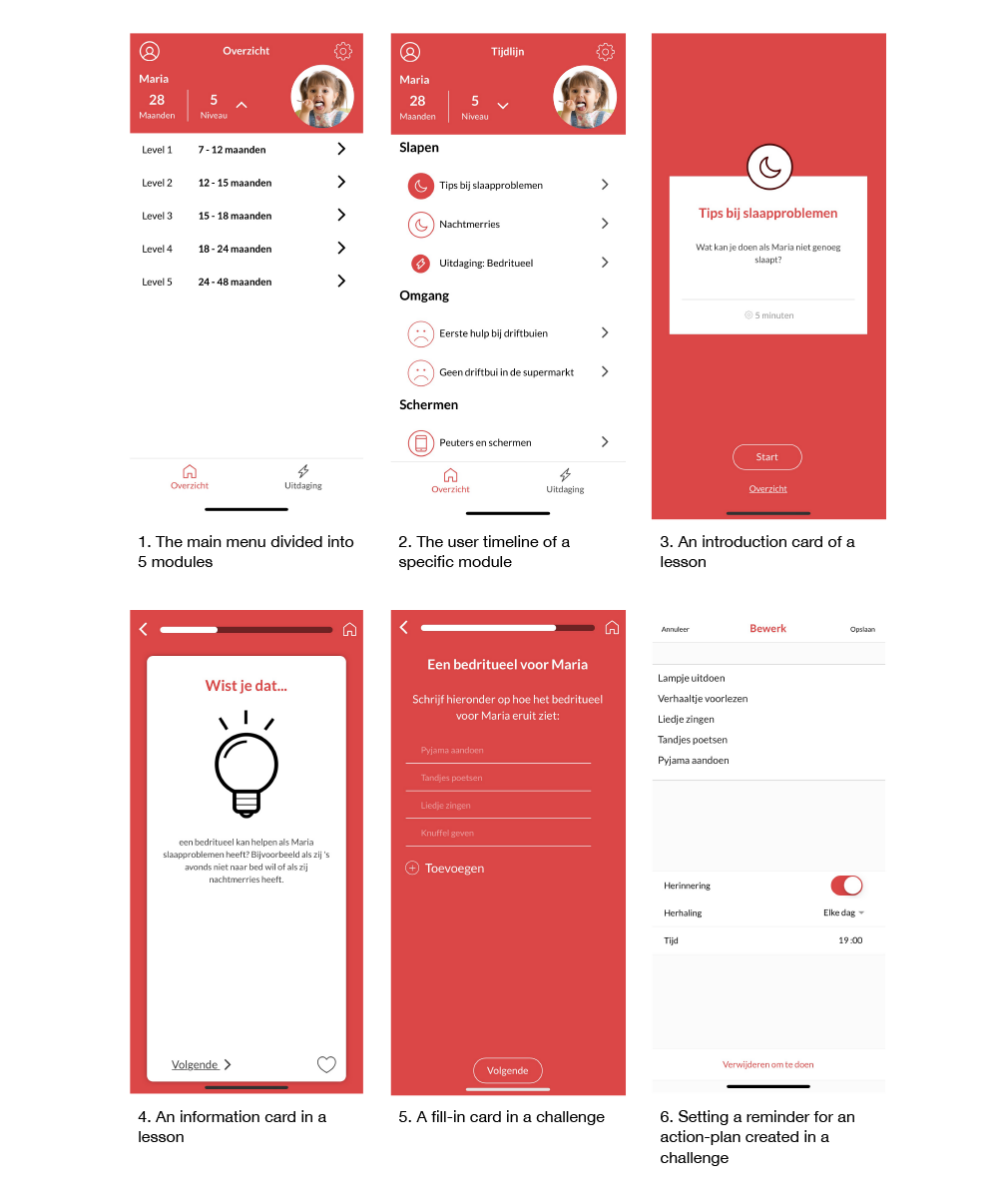
Screenshots of the *Samen Happie!* app showing (1) the main menu divided into 5 modules, (2) the user timeline of a specific module, (3) an introduction card of a lesson, (4) an information card in a lesson, (5) a fill-in card in a challenge, and (6) setting a reminder for an action plan created in a challenge.

The final version of the app (phase 3) was launched in September 2018. Figure 1 presents 6 screenshots of the app (in Dutch). Screenshot 1 shows the main menu of the app. From here, parents could navigate to their profile, app settings, and the actual app content. The content of the app was divided into 5 age-based modules ranging from 7 to 12 months (module 1) to 24 to 48 months (module 5). On the basis of their child’s birth date (entered upon registration), parents were granted access to the appropriate modules (eg, parents of children aged 14 months were granted access to the first 2 modules). When the child reached the minimum age of the subsequent module, the new module became unlocked. The content of each age-based module was divided into 6 themes reflecting all relevant child EBRBs (ie, eating, drinking, sleep, and physical activity and screen time) and themes on the well-being of the parent and temper tantrums (this last theme was only included in the modules for children aged 18 months and older).

Each of the 5 modules consisted of 2 types of activities: lessons and challenges. Screenshot 2 in Figure 1 shows the lessons (indicated by the larger icons) and challenges (indicated by the smaller icons with the thunderstroke) of the themes sleep and temper tantrums in module 5. Upon completion of a lesson or challenge, the icon of that lesson or challenge became filled (see screenshot 2 in Figure 1). The lessons and challenges were constructed using different types of cards. Every lesson or challenge started with an introduction card that showed the title and length of the lesson or challenge (in minutes; see screenshot 3 in Figure 1). The lessons consisted of multiple information cards that presented information on that specific theme in an engaging and easy-to-comprehend manner, for instance, through the use of facts (“Did you know...?”; see screenshot 4 in Figure 1), practical examples, tips, and quizzes and supported by icons or pictures. The challenges consisted of exercises that prompted parents to apply the information from the lessons. By employing techniques that tackle (unhealthy) automatic behaviors, parents were encouraged to implement (new) parenting skills as habits. The challenges were similar in their design. Each challenge started with providing information and examples of specific parenting target behaviors. Next, parents were asked whether they thought performing that behavior was important (ie, attitude) and whether they felt capable (ie, self-efficacy) of performing that behavior. On a slider card, they could indicate their responses on a scale from 0 (low importance or self-efficacy) to 5 (high importance or self-efficacy). Appropriate feedback on their responses was provided through a pop-up notification. Finally, parents created a personal goal or action plan in the context of the target behavior on a fill-in card (see screenshot 5 in Figure 1). For each goal or action plan, parents could set reminders to receive a notification at a date and time of their choice to help fulfill that goal (see screenshot 6 in Figure 1). An illustrated example of a lesson and the accompanying challenge within the theme Sleep can be found in Multimedia Appendix 3. The example includes screenshots of different types of cards within the lesson or challenge, a translation of the original Dutch text to English, and a reference to the theoretical method that formed the basis for that card. Moreover, an overview of the 5 modules and the corresponding lessons and challenges (specified per theme) is presented in Multimedia Appendix 4.

### Step 5: Planning for Adoption, Implementation, and Sustainability

We identified national community health services and child day care centers as potential adopters of the app-based program, as representatives of these organizations were involved in the development and execution of the program. The program implementers will be youth health care professionals and pedagogical staff working at these organizations, and their tasks are to bring the app to the attention of parents (whose children they perceive as being at high risk for obesity) and motivate them to use the app. The implementation of the app-based program can support the daily practice of the program implementers in 2 ways. First, the app could function as an addition to usual care, given that youth health care professionals indicated that standard consultations are generally too brief to give parents elaborate, well-rounded advice. In this sense, it is advantageous that the app is an easy-to-use product that does not require detailed instructions from a health care provider. Second, the app could function as an educational material that creates a legitimate opening to discuss topics such as food parenting and body weight [115], which health professionals often find to be difficult topics to address [67]. In addition, the active role of youth health care professionals in the referral of high-risk groups (in terms of obesity and other health problems) and a primary focus on prevention is in line with the national prevention agreement that the Dutch government issued in the first half of 2019 [116]. Moreover, the agreement calls for the inclusion of (potentially) effective (preventive) interventions for childhood obesity to be registered at the *Healthy Living Desk*, an intervention database of the Dutch ministry of Public Health, Welfare and Sports. To facilitate nationwide dissemination and aid in intervention sustainability, the *Samen Happie!* program was submitted to this database and accepted in September 2020.

### Step 6: Planning the Program Evaluation

The process and effects of the app-based preventive intervention program were evaluated in 2 separate trials, the designs, eligibility criteria, procedures, and measures of which are explained hereafter.

#### Trial Designs

Both trials were randomized controlled trials (RCTs) with 2 parallel arms: an intervention condition in which parents received access to the *Samen Happie!* app and a waitlist control condition. In addition, trial 2 included a third condition in which parents received the app and 2 additional group sessions organized at locations where their child attended preschool. The third condition was a separate condition that did not interfere with the procedures of the RCT, as presented in this manuscript. Detailed information about the development and evaluation of these group sessions has been described elsewhere. Hence, this manuscript will concentrate on the intervention conditions in which parents received access to the app-only and control condition. The parents in both of these conditions completed a web-based baseline measurement (T0) and follow-up measurements at approximately 6 months (T1) and 12 months (T2). Multimedia Appendix 5 provides a schematic overview of the trial flows and includes the exact timing of the measurements.

#### Sample Sizes

A power analysis using G*Power (version 3.1) indicated a minimum of 200 participants in trial 1. This calculation was based on child BMI as the outcome variable, which was assumed to have a mean of 16.67 (SD 1.70) [117]. We further assumed an effect size of 0.20, α of .05, and power of .80. We strived to recruit a minimum of 150 participants per condition (300 in total) to include a minimum of 50% (150/300) of participants with a lower SEP and to account for dropout over time. For trial 2, we recruited 70 participants per condition (140 in total for 2 conditions) based on previous research with a similar design including a face-to-face component [103].

In the end, the participants in trial 1 were 357 parents (346/357, 96.9% biological mothers, 7/357, 1.9% biological fathers, and 4/357, 1.1% nonbiological mothers or partners of the biological mother) of infants aged 5 to 15 months at baseline (ages corresponding to modules 1 and 2 of the app). Trial 2 was conducted among 153 parents (148/153, 96.7% biological mothers, 3/153, 1.9% biological fathers, and 2/153, 1.3% partners of the biological father or mother) with toddlers aged 18 to 55 months at baseline (ages correspond to modules 4 and 5).

#### Eligibility Criteria

To assess whether parents were eligible to participate in the trials, they completed a web-based screening that contained questions about their educational attainment and their child’s age and health status. Parents were respectfully refused participation when their child was younger than 5 months or older than 15 months (trial 1), younger than 20 months or older than 55 months (trial 2), or when their child had a chronic disease or disability that affected normal development. Parents with multiple children could only participate with one child and only in 1 of the 2 trials. We strived to include at least 50% of parents with lower or medium-level SEP and used educational attainment as a proxy for SEP (ie, lower SEP was conceptualized as having completed no education, primary school education, or preparatory vocational education and medium-level SEP was conceptualized as having completed vocational education). Parents with higher educational attainment (ie, preuniversity or university degree) were not actively discouraged from participating in the trials.

#### Procedures

Parents were recruited offline (eg, through child day care centers and community health care centers for young children) and online (eg, through Facebook groups), for which we particularly considered locations that are often visited or used by parents with a lower SEP. Interested parents who fulfilled the eligibility criteria received an email in which they were asked to provide consent for their participation. After consenting, the parents were forwarded to the web-based baseline questionnaire. Randomization for each trial took place after the baseline measurement by means of a simple randomization procedure performed by an independent researcher using SPSS version 24. Among research with large sample sizes, this procedure can be trusted to produce equal samples in terms of numbers and covariates [118]. Participants were compensated for their time and effort with a €10 (US $12) gift card (or a pack of diapers in trial 1) upon completion of the baseline questionnaire and the 2 follow-up measurements. Parents who were allocated to the intervention condition received a personal invitation code for the app to avoid contamination between the 2 conditions. Parents in the intervention condition received instructions on how to download and use the app. There were no instructions regarding the timing and frequency of the use of the app to stay as close as possible to app usage patterns in everyday life. After completing all 3 questionnaires, parents in the control condition were also granted access to the app. The procedures of the trials were approved by the Ethics Committee of the Faculty of Social Sciences, Radboud University, the Netherlands (trial 1: ECSS-2017-013 and trial 2: ECSS-2018-084).

#### Measures

#### Process Evaluation

Two types of process evaluation data were collected: self-reported data and app user data.

#### Parent Self-Reports

We assessed parents’ self-reported app use, their user experience of the app, and their suggestions for app improvement. In the 2 follow-up questionnaires (T1 and T2), we asked parents whether they downloaded the app (and why), whether they still had the app installed on their phone (and why), and how many times they used the app. Regarding user experience, we asked parents to rate several indicators of functionality (eg, ease of use), design, and content (eg, usefulness) on a scale from 1 (bad experience) to 7 (good experience). Parents also rated the app as a whole on a scale from 1 to 10, with higher scores indicating higher appreciation. Finally, we asked open-ended questions about the ways in which the app could be improved.

#### Preliminary Results of Parent Self-Reports

Preliminary analyses of parents’ self-reported app evaluation data on the first follow-up measurement (T1) showed that most parents in the intervention condition of trial 1 (138/179, 77.1% parents) and almost half of the parents in the app-only intervention condition of trial 2 (33/76, 44% parents) reported that they downloaded the app. Most of these parents (127/179, 70.9% in trial 1 and 55/76, 72% in trial 2) indicated that they had used the app multiple times since installation but were not using it anymore at T1. Around a quarter of the parents (39/179, 21.7% in trial 1 and 21/76, 28% in trial 2) indicated that they still used the app multiple times per month. Parents in both trials generally appreciated the functionality, content, and design of the app. They graded the app with an average score of 6.7 (SD 1.45) in trial 1 and 7.2 (SD 1.05) in trial 2. The most important suggestions parents gave for improvement of the app included the incorporation of more detailed and elaborate parenting information, a clearer structure of the presented information (eg, based on weight-related themes instead of age), the option to look for specific information through a search function, and the integration of other parents’ perspectives and experiences (eg, through personal accounts or online interactions).

#### User Data

In addition, to objectively assess parents’ exposure to the preventive intervention program, their app usage was automatically monitored and collected in an online database. This database allowed us to examine the lessons and challenges that parents started and/or finished, the specific lesson cards they saved as *favorite*, and their answers to quiz questions.

#### Effect Evaluation

The primary outcome measures of the effect evaluation were EBRBs of the child (ie, dietary intake, sleep, and screen time), weight-for-height z scores (trial 1), and BMI z scores (trial 2) and parents’ parenting practices related to their child’s EBRBs. The following secondary outcomes were assessed: parents’ general parenting style, parental well-being (ie, depressive symptoms, life satisfaction, stress, and self-reported overall health), and parents’ EBRBs (eg, snacking behavior and sugar-sweetened beverage consumption). An overview of the constructs, variables, and assessment points addressed in the evaluation of the program can be found in Multimedia Appendix 6.

Child weight-for-height and BMI z scores were calculated using height and weight data reported by the parents in the questionnaires. We asked parents to draw this information from the measurement overview in the child’s personal (digital) file, which is updated by the youth health care professional each time the parent and child visit the child health clinic. During the second follow-up questionnaire (T2), we additionally asked parents to send us a picture or screenshot of this measurement overview. This strategy not only allowed us to compare the information parents provided in the questionnaires with that in the child’s file but also allowed us to collect more detailed anthropometric data as the overview contains height and weight measurements from the moment of birth to present day. Moreover, in the second follow-up questionnaire, we asked parents for their permission to be contacted again 12 months and 48 months after T2, so that they could send us a picture or screenshot of the updated measurement overview. This information allowed us to examine the effect of the preventive intervention program on the BMI of the child until approximately 2 years after the last follow-up measurement.

The trials were completed in November 2019 (trial 1) and February 2020 (trial 2). We are currently in the process of data cleaning. Effect analyses are thus underway, and the first results are expected to be submitted for publication in 2021.

## Discussion

### Principal Findings

The need for effective preventive intervention programs for childhood obesity is high, particularly among families with a lower SEP. The app-based parenting program *Samen Happie!* was developed primarily for these families and aims to stimulate healthy energy balance–related parenting practices from early childhood, before unhealthy energy balance–related habits have been established. More specifically, the program promotes both structured and autonomy-supportive practices and limits coercive controlling parenting practices with respect to all relevant energy balance–related determinants of childhood obesity (ie, dietary intake, sleep, physical activity, and screen time), with the ultimate goal of preventing children aged 0 to 4 years old from being overweight and obese. The successful development of the program was aided by the use of the IMP. The process of stepwise decision making made this large-scale and complex project manageable and contributed to thorough considerations. Regarding the selection of eminent EBRBs, for instance, we initially selected only parenting practices related to the children’s dietary intake but we decided to also include practices relating to sleep, physical activity, and screen time after an extensive literature search and discussions with the target group. Moreover, insights from recent literature reviews facilitated the selection of the most promising energy balance–related parenting practices (eg, parental support and modeling for physical activity). By facilitating a collaboration with experts and the target group, the IMP assured that the intended end users of the program were involved in multiple stages of program development. Overall, by integrating theory, empirical studies, professional knowledge, and the needs and preferences of the target group through continuous cocreation, we increased the chances of developing an effective preventive intervention program for childhood obesity [34].

### Strengths, Limitations, and Directions for Future Research

Besides the use of the IMP, the app-based parenting program has several other notable strengths. First, previous digital preventive intervention programs for childhood obesity focused solely on the sociocognitive determinants of energy balance–related parenting [36]. A unique aspect of the *Samen Happie!* program is its focus on both sociocognitive (ie, knowledge, attitudes, and self-efficacy) and automatic (ie, habits) determinants, bridging the well-known gap between health parenting intentions and behaviors [88]. Furthermore, formulating long-term goals is potentially too proximal for parents with a lower SEP as their focus lies primarily on surviving in the here and now [119]. By using a self-regulatory planning approach with personally tailored prompts (eg, through implementation intentions), we facilitated the fulfillment of short-term goals. In addition, by assessing both general parenting style and parental mental well-being as potential moderators of the app-based prevention program, we might be able to identify groups of parents who might particularly benefit from the program, which could contribute to more personalized app usage. Finally, the app is an easy-to-use, stand-alone product that can potentially have a significant reach through its opportunities for widespread implementation.

One of the challenges of mobile health interventions is to keep users engaged for longer periods, which is particularly important for interventions targeting behavior change maintenance [120]. Although the preliminary results of the process evaluation indicated that parents appreciated the functionality, design, and content of the app to a reasonable degree, most parents who downloaded the app did not continue their app use over the course of several months. This might indicate that parents’ information needs were fulfilled and new behavior patterns had been developed, explaining that further app use was no longer needed, but it could also suggest that the user engagement of the app should be increased. This is something that future research should consider. We will further develop the prevention program based on the results of the process evaluation and the input of potential program implementers (eg, youth health care professionals of community health services). On the basis of parents’ suggestions for app improvement, the *Samen Happie!* website [121] has been developed (available in Dutch and English). This website contains more elaborate and structured parenting information and includes a search function, personal accounts of parents, and a forum on which parenting experiences can be exchanged. Future research should examine whether parents’ evaluations of the *Samen Happie!* program improve by offering them access to both the website and the app.

The app-based prevention program also has some limitations. With respect to the design of the trials, it was not possible to blind both participants and investigators to the allocation of conditions (ie, double blinding). As we used a waitlist control condition, the participating parents knew they would eventually receive an app about healthy parenting. However, neither the participants nor the investigators knew which trial condition the participants would be allocated to before randomization took place. Although double blinding in RCTs is generally recommended, methodological studies have shown that adequate allocation concealment is most important in minimizing bias [122,123]. Moreover, regarding our effect evaluations, we were unable to include in-depth measures of the 4 determinants that were targeted in the program (ie, knowledge, attitudes, self-efficacy, and habits), as it was imperative to keep our questionnaires short for our target group of parents with a lower SEP. Nevertheless, we included some questions that could serve as proxies for parental attitudes and self-efficacy. Even though this will give us some indication of the degree to which our preventive intervention program successfully targeted the selected determinants, future research should aim to include detailed measures of its program determinants to be able to examine the working mechanisms of the program.

In addition, future preventive intervention programs for childhood obesity should consider involving both caregivers. Although we intentionally targeted only primary caregivers (who turned out to be primarily mothers) for the recruitment, program materials, and questionnaires of our program, recent research has indicated that parents within a family differ in the energy balance–related parenting practices they perform [124]. This highlights the need for the inclusion of both parents in future preventive intervention programs targeting energy balance–related parenting. Finally, app-based parenting support might not be sufficient for the needs of parents of preschoolers entering their *terrible twos* and food neophobic phase [125,126], especially in the case of parents (with a lower SEP) who already experience parenting problems. To address the more extensive needs of these parents, future research should explore a combination of online tools with additional offline (group based [127]) counseling, which could provide a promising approach to change parenting attitudes and behaviors [128].

### Conclusions

In conclusion, the IMP allowed for effective development of the app-based parenting program *Samen Happie!* to promote healthy energy balance–related parenting practices among parents of infants and preschoolers. By applying the IMP, including continued cocreation, the program specifically addressed the needs of parents with a lower SEP through a tailored program content and through theory-based behavior change techniques. This increases the potential of the program to prevent the development of obesity in early childhood among families with a lower SEP.

## Appendix

Multimedia Appendix 1 Logic model of the health problem addressed in the Samen Happie! program.

Multimedia Appendix 2 Overview of the performance objectives (Tables a-c) and change objectives (Tables d-f) specified for the Samen Happie! program.

Multimedia Appendix 3 Illustrated examples of a lesson and a challenge in the Samen Happie! program.

Multimedia Appendix 4 Overview of the modules and corresponding lessons and challenges of the Samen Happie! app.

Multimedia Appendix 5 Flowchart of the design and timelines for both trials of the Samen Happie! program.

Multimedia Appendix 6 Overview of constructs, variables, and assessment points included in the evaluation of the Samen Happie! program.

## Abbreviations

EBRB: energy balance–related behavior
IMP: Intervention Mapping Protocol
RCT: randomized controlled trial
SEP: socioeconomic position
