# Supplementary figures and images for: An App-Based Parenting Program to Promote Healthy Energy Balance–Related Parenting Practices to Prevent Childhood Obesity: Protocol Using the Intervention Mapping Framework
Source: JMIR Form Res. 2021 May 14;5(5):e24802. doi: 10.2196/24802 (PMC8164123; doi:10.2196/24802)

Logic model of the health problem addressed in the *Samen Happie!* program

*
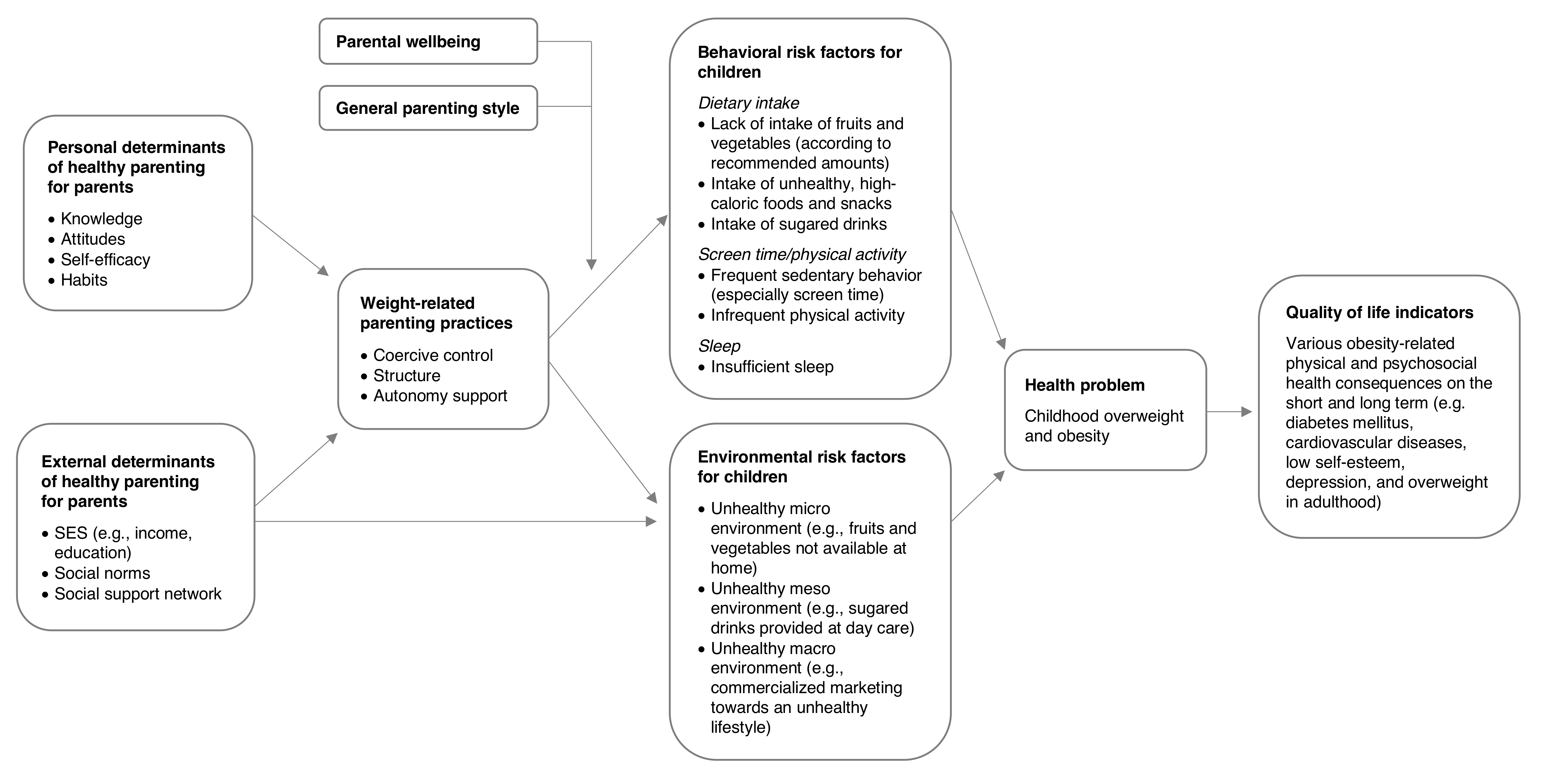
*

Supplement: Multimedia Appendix 1 [file formative_v5i5e24802_app1.docx]
