# Supplementary material for: An App-Based Parenting Program to Promote Healthy Energy Balance–Related Parenting Practices to Prevent Childhood Obesity: Protocol Using the Intervention Mapping Framework
Source: JMIR Form Res. 2021 May 14;5(5):e24802. doi: 10.2196/24802 (PMC8164123; doi:10.2196/24802)
Supplement: Multimedia Appendix 2 [file formative_v5i5e24802_app2.docx]

Overview of the performance objectives (Tables a-c) and change objectives (Tables d-f) of the *Samen Happie!* program

**Table a. Performance objectives for healthy dietary intake**

| **Performance objective** | **Reference to concept map Vaughn et al [10]** |
| --- | --- |
| Parents do not pressure their child to eat or drink. | Pressure |
| Parents do not punish or reward their child with food or drinks. | Threats and bribes |
| Parents apply clear rules about the consumption of healthy and unhealthy food products or drinks. | Rules and limits |
| Parents are consistent with the rules they set about the consumption of healthy and unhealthy food products or drinks. | Rules and limits |
| Parents let their child choose between different healthy food products or drinks (eg, fruit or vegetables, water or tea). | Limited choices |
| Parents monitor the dietary intake of their child. | Monitoring |
| Parents have healthy meal and snack routines (eg, eat at the dinner table, eat at consistent times). | Routines |
| Parents act as a role model by eating or drinking healthy food or drinks themselves. | Modeling |
| Parents make healthy food or drinks available at home. | Availability |
| Parents make healthy food or drinks accessible at home. | Accessibility |
| Parents explain why certain foods or drinks are healthy or unhealthy. | Education |
| Parents involve their child in grocery shopping and preparing healthy food or drinks at home. | Child involvement |
| Parents encourage their child to eat and drink healthy food products or drinks. | Encouragement |
| Parents praise their child when he or she eats healthy food products or drinks water. | Praise |

**Table b. Performance objectives for sufficient sleep**

| **Performance objective** | **Reference to concept map Vaughn et al [10]** |
| --- | --- |
| Parents apply clear rules about bed times. | Rules and limits |
| Parents are consistent in the rules they set about bed times. | Rules and limits |
| Parents make use of bedtime routines. | Routines |
| Parents monitor the sleep duration of their child. | Monitoring |
| Parents ensure a safe and quiet sleep environment for their child. | Accessibility |

**Table c. Performance objectives for restricted screen time and sufficient physical activity**

| **Performance objective** | **Reference to concept map Vaughn et al [10]** |
| --- | --- |
| Parents apply clear rules about screen time. | Rules and limits |
| Parents are consistent with the rules they set about screen time. | Rules and limits |
| Parents monitor the screen time of their child. | Monitoring |
| Parents facilitate activities without the use of screens. | Accessibility |
| Parents act as role models by reducing their use of screens (eg, tablet, television, smartphone) and being physically active themselves. | Modeling |
| Parents explain their child why they have rules about screen time. | Education |
| Parents encourage their child to be physically active (eg, playing outside). | Encouragement |
| Parents praise their child when he or she is doing other activities than engaging in screen time. | Praise |

**Table d. Change objectives for healthy dietary intake by crossing the performance objectives with the selected determinants**

| **Performance objectives** | **Determinants** | | | |
| --- | --- | --- | --- | --- |
|  | Knowledge | Attitudes | Self-efficacy | Habits |
| Parents do not pressure their child to eat or drink. | Parents explain how they can refrain from pressuring their child to eat or drink. | Parents express positive feelings towards refraining from pressuring their child to eat or drink. |  |  |
| Parents do not punish or reward their child with food or drinks. | Parents explain how they can punish or reward their child without the use of food or drinks. | Parents express positive feelings about punishing or rewarding their child without the use of food or drinks. |  |  |
| Parents apply clear rules about the consumption of healthy and unhealthy food products or drinks. | Parents explain how they can apply clear rules about the consumption of healthy and unhealthy food products or drinks. | Parents express positive feelings towards having clear rules for the consumption of healthy food products or drinks. | Parents express confidence in applying clear rules about the consumption of healthy and unhealthy food products or drinks. | Parents consistently apply clear rules about the consumption of healthy and unhealthy food products or drinks. |
| Parents let their child choose between different healthy food products or drinks (eg, fruit or vegetables, water or tea). | Parents explain how they can let their child choose between different healthy food products or drinks. | Parents express positive feelings towards letting their child choose between different healthy food products or drinks. |  |  |
| Parents monitor the dietary intake of their child. | Parents explain how they can monitor the dietary intake of their child. | Parents express positive feelings towards monitoring the dietary behavior of their child. |  |  |
| Parents have healthy meal and snack routines. | Parents sum up how they can apply healthy meal and snack routines. | Parents express positive feelings towards having healthy meal and snack routines. | Parents express confidence in having healthy meal and snack routines. | Parents consistently use healthy meal and snack routines. |
| Parents act as a role model by eating and drinking healthy food or drinks themselves. | Parents explain how they can act as positive role models by eating or drinking healthy food or drinks themselves. | Parents express positive feelings towards acting as a role model by eating or drinking healthy food or drinks themselves. | Parents express confidence in acting as a role model by eating or drinking healthy food or drinks themselves. | Parents consistently act as a role model by eating or drinking healthy food or drinks themselves. |
| Parents make healthy food or drinks available at home. | Parents express how they can make healthy food or drinks available at home. | Parents express positive feelings towards making healthy food or drinks available at home. | Parents express confidence making healthy food or drinks available at home. | Parents consistently make healthy food or drinks available at home. |
| Parents make healthy food or drinks accessible at home. | Parents express how they can make healthy food or drinks accessible at home. | Parents express positive feelings towards making healthy food or drinks accessible at home. | Parents express confidence making healthy food or drinks accessible at home. | Parents consistently make healthy food or drinks accessible at home. |
| Parents explain why certain foods or drinks are healthy or unhealthy. | Parents sum up how they can explain their child why certain foods or drinks are healthy or unhealthy. | Parents express positive feelings towards explaining their child why certain foods or drinks are healthy or unhealthy. |  |  |
| Parents involve their child in grocery shopping and preparing healthy food or drinks at home. | Parents explain how they can involve their child in grocery shopping and preparing healthy food or drinks at home. | Parents express positive feelings towards involving their child in grocery shopping and preparing healthy food or drinks at home. |  |  |
| Parents encourage their child to eat and drink healthy food products or drinks. | Parents tell how they can encourage their child to eat or drink healthy food products or drinks. | Parents express positive feelings about encouraging their child to eat or drink healthy food products or drinks. |  |  |
| Parents praise their child when he or she eats healthy food products or drinks water. | Parents explain how they can praise their child when he or she eats healthy food products or drinks water. | Parents express positive feelings towards praising their child when he or she eats healthy food products or drinks water. |  |  |

Note. Not all performance objectives were translated into change objectives, as indicated by the empty cells in the table.

**Table e. Change objectives for sufficient sleep by crossing the performance objectives with the selected determinants**

| **Performance objectives** | **Determinants** | | | |
| --- | --- | --- | --- | --- |
|  | *Knowledge* | *Attitudes* | *Self-efficacy* | *Habits* |
| Parents apply clear rules about bed times. | Parents express how they can apply clear rules about bed times. | Parents express positive feelings towards applying clear rules about bed times. |  |  |
| Parents are consistent in the rules they set about bed times. | Parents explain how they can be consistent in the rules they set about bed times. | Parents express positive feelings about being consistent in the rules they set about bed times. |  |  |
| Parents make use of bedtime routines. | Parents explain how they can make use of bedtime routines. | Parents express positive feelings towards making use of bedtime routines. | Parents express confidence about making use of bedtime routines. | Parents consistently make use of bedtime routines. |
| Parents monitor the sleep duration of their child. | Parents explain how they can monitor the sleep duration of their child. | Parents express positive feelings about monitoring the sleep duration of their child. |  |  |
| Parents ensure a safe and quiet sleep environment for their child. | Parents express how they can ensure a safe and quiet sleep environment for their child. | Parents express positive feelings about ensuring a safe and quiet sleep environment for their child. |  |  |

Note. Not all performance objectives were translated into change objectives, as indicated by the empty cells in the table.

**Table f. Change objectives for restricted screen time and sufficient physical activity by crossing the performance objectives with the selected determinants**

| **Performance objectives** | **Determinants** | | | |
| --- | --- | --- | --- | --- |
|  | *Knowledge* | *Attitudes* | *Self-efficacy* | *Habits* |
| Parents apply clear rules about screen time. | Parents explain how they can apply clear rules about screen time. | Parents express positive feelings towards applying clear rules about screen time. | Parents express confidence in applying clear rules about screen time. | Parents consistently apply clear rules about screen time. |
| Parents monitor the screen time of their child. | Parents explain how they can monitor the screen time of their child. | Parents express positive feelings towards monitoring the screen time of their child. |  |  |
| Parents facilitate activities without the use of screens. | Parents explain how they can facilitate activities without the use of screens. | Parents express positive feelings towards facilitating activities without the use of screens. | Parents express confidence in facilitating activities without the use of screens. | Parents consistently facilitate activities without the use of screens. |
| Parents act as role models by reducing their use of screens (eg, tablet, television, smartphone) and being physically active themselves. | Parents explain how they can act as role models by reducing their use of screens and being physically active themselves. | Parents explain positive feelings towards acting as role models by reducing their use of screens and being physically active themselves. |  |  |
| Parents explain their child why they have rules about screen time. | Parents tell how they can explain their child why they have rules about screen time. | Parents express positive feelings towards explaining their child why they have rules about screen time. |  |  |
| Parents encourage their child to be physically active (eg, playing outside). | Parents explain how they can encourage their child to be physically active. | Parents express positive feelings towards encouraging their child to be physically active. |  |  |
| Parents praise their child when he or she is doing other activities than engaging in screen time. | Parents explain how they can praise their child when he or she is doing other activities than engaging in screen time. | Parents express positive feelings towards praising their child when he or she is doing other activities than engaging in screen time. |  |  |

Note. Not all performance objectives were translated into change objectives, as indicated by the empty cells in the table.
