# Supplementary material for: An App-Based Parenting Program to Promote Healthy Energy Balance–Related Parenting Practices to Prevent Childhood Obesity: Protocol Using the Intervention Mapping Framework
Source: JMIR Form Res. 2021 May 14;5(5):e24802. doi: 10.2196/24802 (PMC8164123; doi:10.2196/24802)
Supplement: Multimedia Appendix 3 [file formative_v5i5e24802_app3.pdf]

Illustrated examples of a lesson and a challenge

On the following pages, examples are provided of the two main activities of the *Samen Happie!* app: following a lesson and doing a challenge.

Each example shows six screenshots of different cards in that lesson/challenge. Below each screenshot, a description of the card in English is provided. Moreover, the behavior change technique (BCT) or techniques that form the basis of that card are specified. For an overview of all BCTs included in the program and their descriptions, please consult Table 2 in the manuscript.

The examples described here are from the theme Sleep of Module 5 (24-48 months). The screenshot on the right shows this theme in the user timeline. The lesson (indicated by the larger filled icon) is called Tips for sleep problems. The main goal of this lesson is to provide parents with practical tips and guidelines on how to behave when their child does not get sufficient sleep. The challenge (indicated by the smaller filled icon with the thunderstrike) that accompanies this lesson is called Creating a bedtime ritual. The main goal of this challenge is to prompt parents to create a bedtime routine and stimulate the habitual use of this routine.

The lesson and challenge illustrated here correspond to the performance objective: Parents make use of bedtime routines (from Table 1 of the manuscript). More specifically, the lesson and challenge together address the following change objectives for child sleep:

- Parents explain how they can make use of bedtime routines (determinant Knowledge)
- Parents express positive feelings towards making use of bedtime routines (determinant Attitudes)
- Parents express confidence in making use of bedtime routines (determinant Self-efficacy)
- Parents consistently make use of bedtime routines (determinant Habits)

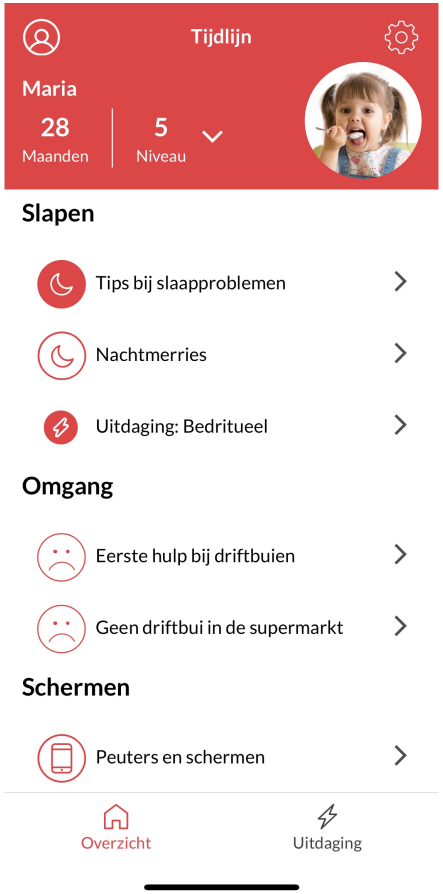

Lesson - Tips for sleep problems

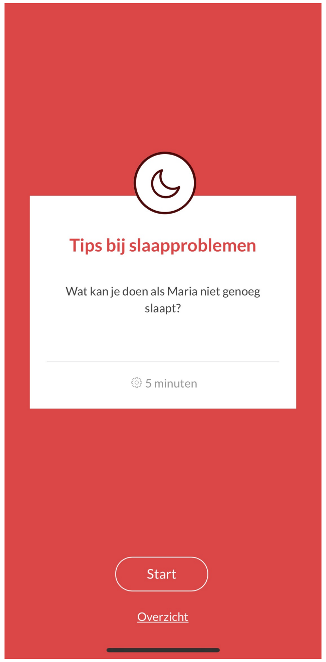

This introduction card shows the title (Tips for sleep problems) and the length of the lesson (5 minutes).

BCT
-

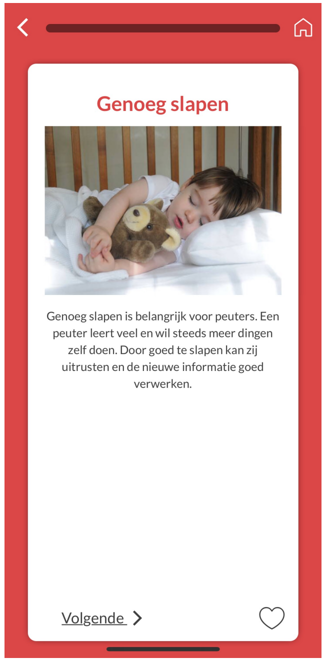

This information card explains the benefits of sufficient sleep for toddlers: that it is crucial to rest and be able to process all new information that comes with being a toddler.

BCT
Consciousness raising, framing

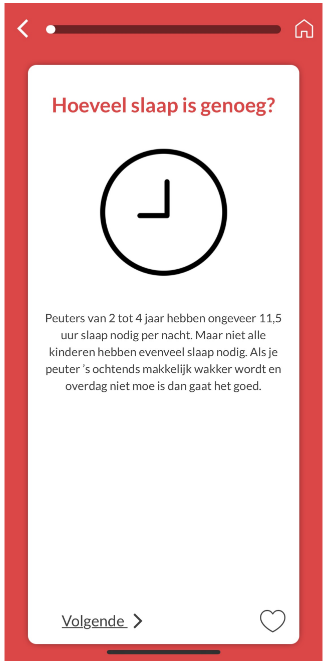

This information card explains how many hours of sleep a toddler (2-4 years) needs: around 11,5 hours per night, but this may vary per child. To check whether a child is rested, it is best to check whether he/she gets up easily in the morning and is not tired during the day.

BCT
Consciousness raising, instruction how to perform a behavior

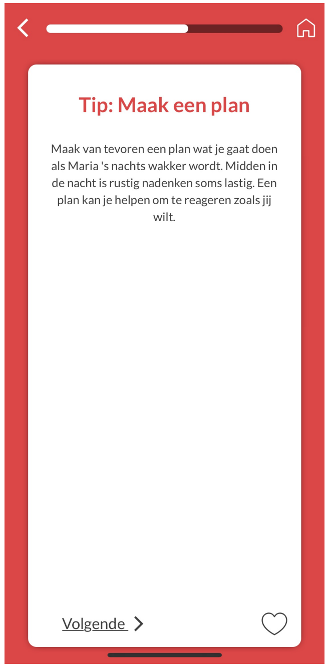

This information card advises parents to make an if-then plan on how they want to act when their child wakes up in the middle of the night. The following card (not shown here) provides an example of an if-then plan.

BCT
Implementation intention

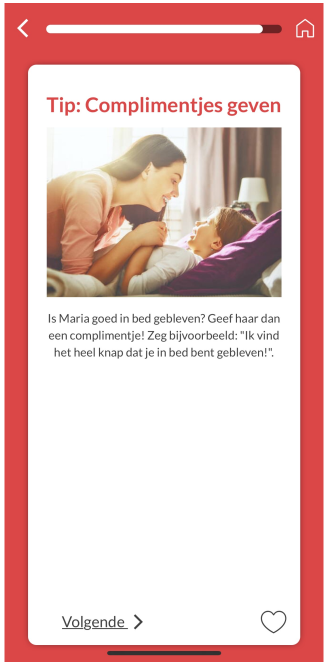

This information card advises parents to compliment their child when he/she stayed in bed all night and gives an example: “I’m so proud of you for staying in your bed!”

BCT
Instruction how to perform a behavior

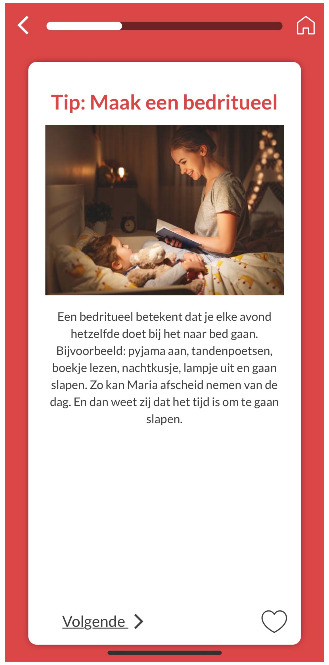

This information card advises parents to make a bedtime ritual for their child, explains what a bedtime ritual is and gives an example of a bedtime ritual.

BCT
Instruction how to perform a behavior

Challenge - Creating a bedtime routine

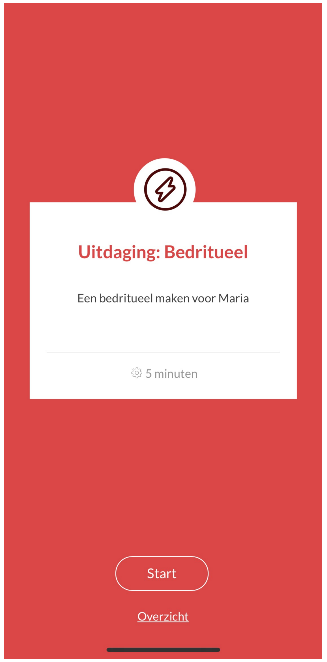

This introduction card shows the title (Creating a bedtime ritual) and the length of the challenge (5 minutes).

BCT
-

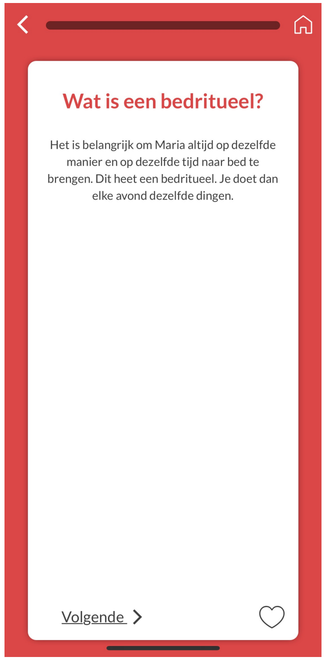

This information card explains what a bedtime ritual is (doing the same things every night in the same order, before it is time to sleep) and that this ritual is important.

BCT
Consciousness raising

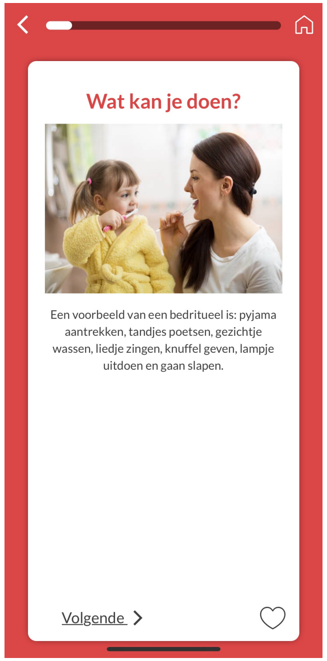

This information card gives an example of a bedtime routine (eg, putting on pajamas, brushing teeth, wash face, sing a little song, give a hug, turn off the light, go to sleep).

BCT
Instruction on how to perform a behavior

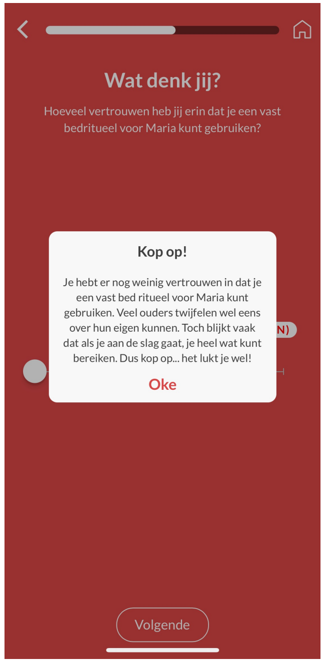

This slider card asks parents to indicate their self-efficacy towards creating a bedtime routine for their child on a scale from 0 (low) to 5 (high). Because the answer was 1, the pop-up notification shows an encouraging message that stimulates parents to try to make the bedtime routine.

BCT
Verbal persuasion

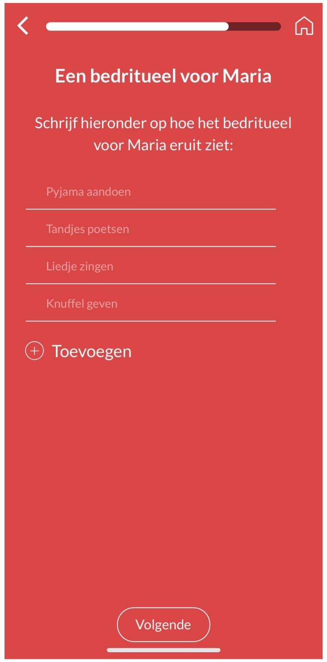

This fill-in card asks parents to describe their new step-by-step bedtime routine. On the card that follows (not displayed here), it is optional to set a reminder for this action by creating a notification for specific days and times.

BCT
Action planning

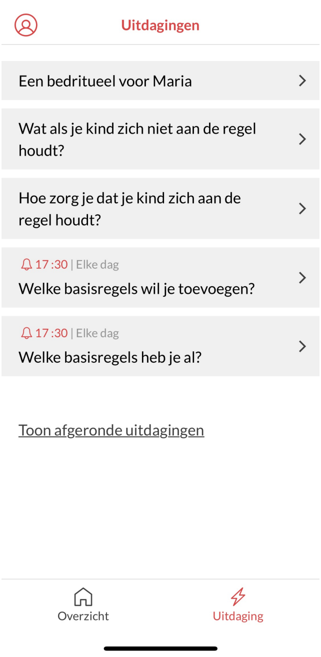

All goals and plans parents made in the challenges (including the reminders they set) are displayed together in one place.

BCT
-
