# Supplementary material for: An App-Based Parenting Program to Promote Healthy Energy Balance–Related Parenting Practices to Prevent Childhood Obesity: Protocol Using the Intervention Mapping Framework
Source: JMIR Form Res. 2021 May 14;5(5):e24802. doi: 10.2196/24802 (PMC8164123; doi:10.2196/24802)
Supplement: Multimedia Appendix 4 [file formative_v5i5e24802_app4.docx]

Overview of the modules and corresponding lessons and challenges of the *Samen Happie!* app

|  |  | **Titles of the lessons and challenges per theme** | | | | | |
| --- | --- | --- | --- | --- | --- | --- | --- |
| **Module** | **Child age** | Eating | Drinking | Sleeping | Screen time | Wellbeing | Tantrums |
| 1 | 7 – 12 months | Introducing solid foods, learning to eat new flavors (lesson + challenge), tips to introduce new foods, how much food is enough | How to give your child water, practicing with drinking water from a cup (challenge), why drinking water is important, how to read food labels | How much sleep is enough, how to create a bedtime ritual (lesson + challenge) |  | Taking care of yourself as a parent, tips to create time for yourself |  |
| 2 | 12 – 15 months | Snacking, why it is easy to consume unhealthy food and drinks, creating a healthy home environment (challenge) | Tips to give your child water (instead of sugared drinks), drinking water (challenge) |  | Setting limits for screen time, suggestions for offline activities, how to get sufficient physical activity (challenge) | Positive thinking, tips to think positive thoughts |  |
| 3 | 15 – 18 months | Eating and control, giving a good example (lesson + challenge) | Creating a healthy drink environment, reasons for choosing water over sugared drinks | When your child does not want to go to sleep (lesson + challenge), creating a healthy sleep environment |  | Parenting as a team effort, how to discuss parenting rules with others |  |
| 4 | 18 – 24 months | Consistent mealtime routines, tips for having a family dinner at the table (lesson + challenge), strategies to apply when your child refuses to eat | Tips to offer healthy drinks, common myths about (healthy) drinks | Tips to get enough sleep | Setting rules about screen time (lesson + challenge), getting enough physical activity | What is stress, how to reduce stress, ways to prevent stress | How to deal with temper tantrums |
| 5 | 24 – 48 months | Having rules about food, creating food rules (challenge), how to stimulate healthy food intake, what to do when your child refuses to eat vegetables, options to healthy with little time and money. | Drinking water, tips to give your child water, drinking water with meals, why packaging labels do not always tell the truth. | Tips for sleep problems, how to deal with nightmares, creating a bedtime ritual (challenge) | Toddlers and screens, media suggestions for toddlers | Mindfulness, mindfulness exercises, mindful parenting | How to deal with temper tantrums, ways to prevent a temper tantrum at the grocery store |

Note. All information described in the main themes concern the child’s behavior, except for the theme “Wellbeing”, which provides information about parents’ mental health.
