# Supplementary material for: An App-Based Parenting Program to Promote Healthy Energy Balance–Related Parenting Practices to Prevent Childhood Obesity: Protocol Using the Intervention Mapping Framework
Source: JMIR Form Res. 2021 May 14;5(5):e24802. doi: 10.2196/24802 (PMC8164123; doi:10.2196/24802)
Supplement: Multimedia Appendix 5 [file formative_v5i5e24802_app5.docx]

Flow chart of the design and timelines for both trials of the *Samen Happie!* program

Intervention group (app)

*N* = 179 for Trial 1 | *N* = 76 for Trial 2

Control group (waitlist)

*N* = 178 for Trial 1 | *N* = 77 for Trial 2

January – August 2018

June – August 2018

September 2018

December 2018 – January 2019

July – November 2019

Timeline Trial 1

Timeline Trial 2

March – November 2018

September – November 2018

November 2018

April – May 2019

August 2019 – February 2020

Eligibility screening

Recruitment of parent-child dyads

*N* = 485 for Trial 1 | *N* = 233 for Trial 2

Invitation baseline measurement (T0)

*N* = 473 for Trial 1 | *N* = 201 for Trial 2

Randomization

*N* = 357 for Trial 1 | *N* = 153 for Trial 2

Excluded (*N* = 12)

- Did not meet criteria (*n* = 5)
- Did not consent (*n* = 1)
- Multiple registrations (*n* = 6)

First follow-up (T1)

*N* = 162 for Trial 1 | *N* = 66 for Trial 2

First follow-up (T1)

*N* = 171 for Trial 1 | *N* = 72 for Trial 2

Second follow-up (T2)

*N* = 159 for Trial 1 | *N* = 67 for Trial 2

Second follow-up (T2)

*N* = 154 for Trial 1 | *N* = 61 for Trial 2

Excluded (*N* = 116)

- Invalid e-mail (*n* = 2)
- Unable to login (*n* = 1)
- No response (*n* = 110)
- Did not finish baseline (*n* = 3)

Excluded (*N* = 48)

- Invalid e-mail (*n* = 3)
- No response (*n* = 30)
- Did not finish baseline (*n* = 15)

Excluded (*N* = 32)

- Did not meet criteria (*n* = 14)
- Duplicate registration Trial 1 (*n* = 17)
- Multiple registrations (*n* = 1)
