# Supplementary material for: An App-Based Parenting Program to Promote Healthy Energy Balance–Related Parenting Practices to Prevent Childhood Obesity: Protocol Using the Intervention Mapping Framework
Source: JMIR Form Res. 2021 May 14;5(5):e24802. doi: 10.2196/24802 (PMC8164123; doi:10.2196/24802)
Supplement: Multimedia Appendix 6 [file formative_v5i5e24802_app6.docx]

Overview of constructs, variables, and assessment points included in the evaluation of the *Samen Happie!* program

|  | | | **Assessment Points** | | | | | |
| --- | --- | --- | --- | --- | --- | --- | --- | --- |
|  |  |  | Trial 1 | | | Trial 2 | | |
| **Domain** | **Constructs** | **(Examples of) Variables** | T0 | T1 | T2 | T0 | T1 | T2 |
| Meta data | Questionnaire meta data | Questionnaire start date, end date, duration | x | x | x | x | x | x |
| Demographics and anthropometry | Demographics child | Date of birth, sex, country of birth, birth weight, current height, current height | x | x | x | x | x | x |
|  | Demographics parent | Relationship to child, country of birth, birth date, current height, current weight, educational attainment, occupational status, financial difficulty, zip code | x | x | x | x | x | x |
|  | Family characteristics | Number of children in household, age of children, relationship status, duration of relationship | x | x | x | x | x | x |
|  | Demographics partner | Relationship to child, country of birth, birth date, current height, current weight, educational attainment, occupational status | x | x | x | x | x | x |
| Energy balance-related behaviors child | Beverages consumption | Breastfeeding, consumption of water, tea, (light) juice, (skimmed/sugared) dairy | x | x | x | x | x | x |
|  | Bread and porridge consumption | Consumption of bread and porridge |  | x | x |  | x | x |
|  | Fruit consumption | Consumption of ready-made fruit meals and fresh fruit | x | x | x | x | x | x |
|  | Snack consumption | Consumption of crackers, biscuits, cookies (small/large) | x | x | x | x | x | x |
|  | Vegetables consumption | Consumption of ready-made meals, fresh vegetables, vegetables as snack | x | x | x | x | x | x |
|  | Eating behavior | Food fussiness, food responsiveness | x | x | x | x | x | x |
|  | Screen time | Screen time (during meals) | x | x | x | x | x | x |
|  | Sleep | Sleep onset, waking times, sleep duration (day and night) | x | x | x | x | x | x |
| Parenting | Parenting practices related to beverages consumption | Rules about sugared drinks | x | x | x | x | x | x |
|  | Parenting practices related to food consumption | Autonomy supportive practices (eg, nutrition education, praise, encouragement), structured practices (eg, rules and limits, availability, routines), coercive controlling practices (eg, emotional feeding, instrumental feeding) | x | x | x | x | x | x |
|  | Parenting practices related to screen time | Rules about screen time | x | x | x | x | x | x |
|  | Parenting practices related to sleep | Sleep routines | x | x | x | x | x | x |
|  | General parenting style | Nurturance, structure, behavioral control, coercive control, overprotection |  | x | x | x | x | x |
| Wellbeing parent | Perceived stress | Perceived stress at home, at work, about parenting | x | x | x | x | x | x |
|  | Depressive symptoms | Depressive symptoms | x | x | x | x | x | x |
|  | Parenting self-efficacy | Parenting self-efficacy | x | x | x | x | x | x |
|  | Life satisfaction | Life satisfaction | x | x | x | x | x | x |
|  | Social support | Social support |  | x | x | x | x | x |
|  | Perceived health | Perceived emotional health, perceived physical health | x | x | x | x | x | x |
| Energy balance-related behaviors parent | Food intake and dieting | Snacking, dieting, food addiction, beverages consumption | x | x | x | x | x | x |
|  | Substance use | Alcohol consumption, smoking | x | x | x | x | x | x |
|  | Screen time | Screen time, app use | x | x | x | x | x | x |
| Program evaluation | Recruitment | Recruitment medium (eg, online, via flyer, through health professional or child day care) | x |  |  | x |  |  |
|  | App | Downloaded, currently installed, frequency of use, user experience (eg, functionality, design, content), rating |  | x | x |  | x | x |
